# adipoSIGHT in Therapeutic Response: Consequences in Osteosarcoma Treatment

**DOI:** 10.3390/bioengineering8060083

**Published:** 2021-06-10

**Authors:** Banani Kundu, Virginia Brancato, Joaquim Oliveira, Vitor M. Correlo, Rui L. Reis, Subhas C. Kundu

**Affiliations:** 1 3B’s Research Group, I3Bs—Research Institute on Biomaterials, Biodegradables and Biomimetics, University of Minho, Headquarters of the European Institute of Excellence on Tissue Engineering and Regenerative Medicine, AvePark, Parque de Ciência e Tecnologia, Zona Industrial da Gandra, 4805-017 Guimarães, Portugal; brancatovirginia@gmail.com (V.B.); miguel.oliveira@dep.uminho.pt (J.O.); vitorcorrelo@i3bs.uminho.pt (V.M.C.); rgreis@i3bs.uminho.pt (R.L.R.); 2 ICVS/3B’s—PT Government Associate Laboratory, 4805-017 Guimarães, Portugal

**Keywords:** heterotypic tumorspheres, nonadherent surface, osteosarcoma, human adipose-derived stem cells, drug response

## Abstract

Chemotherapeutic resistance is a major problem in effective cancer treatment. Cancer cells engage various cells or mechanisms to resist anti-cancer therapeutics, which results in metastasis and the recurrence of disease. Considering the cellular heterogeneity of cancer stroma, the involvement of stem cells is reported to affect the proliferation and metastasis of osteosarcoma. Hence, the duo (osteosarcoma: Saos 2 and human adipose-derived stem cells: ASCs) is co-cultured in present study to investigate the therapeutic response using a nonadherent, concave surface. Staining with a cell tracker allows real-time microscopic monitoring of the cell arrangement within the sphere. Cell–cell interaction is investigated by means of E-cadherin expression. Comparatively high expression of E-cadherin and compact organization is observed in heterotypic tumorspheres (Saos 2–ASCs) compared to homotypic ones (ASCs), limiting the infiltration of chemotherapeutic compound doxorubicin into the heterotypic tumorsphere, which in turn protects cells from the toxic effect of the chemotherapeutic. In addition, genes known to be associated with drug resistance, such as SOX2, OCT4, and CD44 are overexpressed in heterotypic tumorspheres post-chemotherapy, indicating that the duo collectively repels the effect of doxorubicin. The interaction between ASCs and Saos 2 in the present study points toward the growing oncological risk of using ASC-based regenerative therapy in cancer patients and warrants further investigation.

## 1. Introduction

Osteosarcoma is the most common primary tumor in children and adolescents. Approximately 15–20% of osteosarcoma patients demonstrate clinically detectable metastasis with poorer prognosis [1]. The 5-year survival rate is less than 70% with recurring metastasis and drug resistance [2]. The multimodal treatment of osteosarcoma includes chemotherapy and surgical intervention [3]. Despite the development of several strategies, the reconstruction of bone and soft tissue defects after resection remains a critical challenge [3]. In clinical situations where healing is impaired due to insufficient supply of blood or chronic inflammation in such sites after radiotherapy, the use of human adipose stem cells (ASCs) is encouraged due to their multipotent nature. After the use of autologous fat grafts in reconstruction surgery post-chemotherapy, patients have reported the recurrence of osteosarcoma [4]. Our previous work has shown the formation of a more aggressive osteosarcoma (Saos 2) tumorsphere in the presence of ASCs, indicating the regulatory role of hetero-cellular tumor stroma in cancer progression [5]. Hence, the governing role of ASCs in the therapeutic response and resistance, especially in cancer, needs to be fully understood.

The oncological risk in using the autologous ASCs in reconstruction surgery post-chemotherapy has already been reported [6,7]. Unfortunately, the conventional 2D culture platforms are employed in these models, which have poor reliability due to the absence of the spatial cellular arrangement. The penetration of drugs within 3D spatially arranged cells is significantly different from its 2D counterparts, which affects the clinical outcome. Hence, to gain an insight into the role of ASCs in the therapeutic response of osteosarcoma and their subsequent involvement in the development of therapeutic resistance in the clinic, in this study, ASCs are co-cultured with Saos 2 using agar-based nonadherent culture plates. The co-cultures are investigated for the formation and stability of the 3D tumorspheres over time. The expression of genes is further analyzed and compared with their homotypic counterparts for up/downregulation in order to understand the effect of hetero-cellular crosstalk. In this way, an in vitro cancer model is established that mimics the influence of mesenchymal stem cells (ASCs) on mesenchymal carcinoma cells (Saos 2). Visible doxorubicin resistance is obtained in osteosarcoma cells co-cultured with adipose stem cells (Saos 2–ASCs). Therefore, we have further examined the correlation between co-cultured Saos 2–ASCs and chemoresistance using an in vitro tumorsphere model. An understanding of the underlying mechanisms of tumor-encouraging chemoresistance activated by ASCs in cancer cells may augment the list of potential targets for therapeutic management while overcoming challenges such as chemoresistance in osteosarcoma.

## 2. Materials and Methods

### 2.1. Preparation of Agarose-Coated Wells

**Scheme 1:**
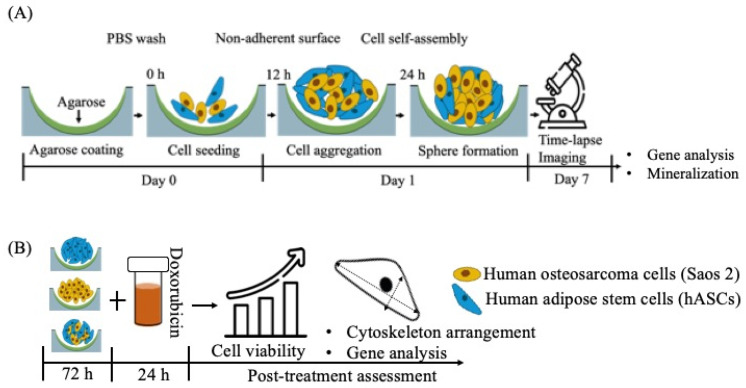
Schematics of (**A**) experimental outline; (**B**) chemotherapeutic screening.

Agarose (SeaKem LE AGAROSE 500G, Lonza, Rockland, USA of analytical grade) was dissolved at 90 °C by stirring in deionized water (1 wt%) and sterilized at 121 °C for 20 min in an autoclave [8]. It was then left to cool down to 70 °C. Approximately 60 μL of the liquid was pipetted into each well of a 96-well plate and was allowed to solidify at room temperature under a sterile hood (Scheme 1). The wells were then washed with phosphate-buffered saline (PBS, pH 7.4, Sigma).

### 2.2. Cell Isolation and Culture

Human ASCs were collected from patients at Hospital da Prelada (Porto, Portugal) after liposuction and processed at 3B’s Research Group following the ethical guidelines approved by both the institutions [9]. Prior to use, the isolated ASCs were then characterized phenotypically and their multipotency/stemness was confirmed by their ability to differentiate into different lineages. The cultured cells (*p* = 3) were investigated for ASC-specific surface markers including CD105-FITC, CD73-PE, CD90-APC, CD45-FITC, CD34-PE, and CD31-APC (BD Biosciences, Franklin Lakes, NJ, USA) using flow cytometry. Briefly, the cells after trypsinization were resuspended in BSA (3%, Sigma-Aldrich, St. Louis, MO, USA) solution and left at room temperature for 30 min, followed by incubation with mouse anti-human antibodies following manufacturers’ recommendations. After staining, the cells were rinsed with PBS (Sigma, Louis, MO, USA), fixed in paraformaldehyde (1% *v*/*v*), analyzed using the FACS Aria III (BD Biosciences, Franklin Lakes, NJ, USA), and then analyzed with Diva Software (BD Bioscience, Franklin Lakes, NJ, USA). Approximately 20,000 events per tube were read.

The adipogenic and osteogenic differentiation of ASCs were induced by treating the cells using the respective media cocktail. Approximately 1 × 10^5^ cells/cm^2^ (*p* = 3) were seeded in a culture plate and treated with adipogenic (αMEM containing 1 μM dexamethasone, 0.5 mM 1-methyl-3-isobutylxanthine, 1 ng/mL insulin, and 100 μM indomethacin) [10] and osteogenic (αMEM supplemented with 10 mM β-glycerol phosphate, 10 mM dexamethasone, and 50 μg/mL ascorbic acid) [9] media for 21 days.

Saos 2 cells were purchased from the European Collection of Cell Cultures and maintained in αMEM supplemented with 10% fetal bovine–1% penicillin/streptomycin. For sphere culture, cells were seeded at a density of 5 × 10^4^ cells/well (1:1 ratio in heterotypic culture) (Scheme 1A).

### 2.3. 3D Time-Lapse Confocal Microscopy

After trypsinization, the cells were rinsed with serum-free media and incubated with dye (1x) prepared in Diluent C provided in the kit (Sigma-Aldrich, St. Louis, MO, USA) for 5 min; ASCs were labelled with PKH26 Red Fluorescent Cell Linker and Saos 2 with PKH67 Green Fluorescent Cell Linker, respectively [5]. The cells were then seeded on an agarose-coated plate and maintained under standard culture conditions (in αMEM containing 10% fetal bovine–1% penicillin/streptomycin). The self-assembly of cells was imaged under a confocal microscope, TCS SP8 (Leica Microsystems, Wetzlar, Germany), at 490 nm (PKH67) and 551 nm (PKH26) excitations in a time-lapse series for one week (Scheme 1A).

### 2.4. Calcium Depletion Test

The calcium depletion test was performed following the standard protocol described elsewhere [11]. Briefly, the medium of the stable 3D tumorsphere after 72 h of culture was replaced with serum-free medium containing 4 mmol/L EDTA and incubated for 30 min. The tumorspheres were then subjected to trypsin digestion for 10 min without EDTA. Cell spheres which were only subjected to trypsin digestion (10 min) without any EDTA treatment were used as controls. The segmentation of the tumorspheres was then observed under a microscope (Leica DM750, Wetzlar, Germany) with an MRc5 camera.

### 2.5. Mineralization Assay

The mineralization was induced by culturing the cells using differentiation medium (αMEM supplemented with 10% fetal bovine serum–1% antibiotic–10 mM β-glycerol phosphate and 50 μg/mL ascorbic acid) for up to 14 days [12]. The medium was changed every other day. After the completion of the culture, the mineralized tumorspheres were fixed in formalin (10% *v*/*v*, 30 min at room temperature), dehydrated in a graded ethanol series (30–100% *v*/*v*), and the elemental analysis was carried out using FE-SEM (JEOL JSM 6301F/Oxford INCA Energy 350/Gatan Alto 2500) coupled with energy-dispersive X-ray spectroscopy (EDS). The high-energy electrons impact the atoms of the sample surface. Some of these atoms become ionized. When they return to their ground state, the atoms emit X-rays that are unique to that element. The X-rays are akin to the fingerprint for that element. The EDS detector detects the X-rays and measures both the frequencies and the corresponding intensities. The frequencies provide information on the type of elements present on the sample surface, while the intensities provide information on the concentration of each element. The samples were sputter-coated with platinum (3 nm) and scanning electron micrographs were collected at an operating voltage of 3 kV.

### 2.6. Cytotoxicity Assay

The effect of doxorubicin (DOX, Carbosynth Ltd., Berkshire, UK) on the viability of tumorspheres was evaluated by MTS assay (Scheme 1B). The cells, after seeding (5 × 10^4^ cells/well), were allowed to grow for 3 days and then treated with diluted doxorubicin (5–1 μg/mL) for 24 h, followed by MTS assay (3-(4,5-dimethylthiazol-2-yl)-5-(3-carboxymethoxyphenyl)-2-(4-sulfophenyl)-2H-tetrazolium (MTS, Promega, Madison, WI) as per the manufacture’s protocol. Briefly, after treatment, the cell spheres were incubated with MTS assay reagent for 2 h in the dark at 37 °C in a 5% CO_2_ atmosphere. After the incubation was over, the absorbance of the MTS reagent was measured using a microplate spectrophotometer (Synergy HT, Bio-Tek, Winooski, VT, USA) at 490 nm. The viability of cells was represented as percentages with reference to the control (untreated cell spheres).

### 2.7. Actin Staining

The cell spheres were fixed in formalin (10% *v*/*v*, Enzifarma, Lisboa, Portugal) [13]. The actin filaments were stained using Phalloidin–tetramethylrhodamine B isothiocyanate (1:500 *v*/*v* in PBS, Sigma) and nuclei with DAPI [(4.6-diamidino-2-phenylidole)] (1:1000 *v*/*v* in PBS, Sigma). The images were taken under an inverted confocal microscope (TCS SP8, Leica Wetzlar, Germany). For DAPI, fluorescence wavelengths of 415 nm and 461 nm were used for excitation and emission, respectively. Regarding Phalloidin, an excitation wavelength of 560 nm and an emission wavelength of 630 nm were used. Images were acquired with 20x water and 63x oil objectives.

### 2.8. Gene Expression Analysis

Total mRNA was extracted using TRI Reagent^®^ RNA Isolation Reagent (ThermoFisher Scientific, Waltham, MA, USA), according to the manufacturer’s guidelines. RNA concentration and purity were measured by means of a Nanodrop^®^ ND-1000 spectrophotometer (ThermoFisher Scientific, Waltham, MA, USA) [5]. The cDNA synthesis was performed on 200 ng of mRNA using the qScriptTM cDNA Synthesis Kit (Quanta BioSciences, Gaithersburg, MD, USA). Briefly, a reaction mixture consisting of 4 μL qScript Reaction Mix, 1 μL qScript Reverse Transcriptase, RNA template (200 ng total RNA), and nuclease-free water was calculated considering a final volume of 20 μL. The single-strand cDNA was synthesized by incubating the complete reaction mixture for 5 min at 22 °C, followed by 30 min at 42 °C, ending with an incubation of 5 min at 85 °C. RT-PCR was performed using PerfeCTA^®^ SYBR Green FastMix (Quanta BioSciences, Gaithersburg, MD, USA), following the manufacturer’s instructions, on the RT-PCR Mastercycler Realplex machine (Realplex, Eppendorf, Germany). Primer sequences (Eurofins Genomics, 23 Val Fleuri, Luxembourg) were designed using the Primer-BLAST tool (Table 1). Relative gene expression was obtained using Livak’s method (2−ΔΔCt) [14]. The mRNA expression was first normalized to the average expression of an endogenous control gene such as glyceraldehyde-3-phosphate dehydrogenase (GAPDH). Three samples of each type were considered.

### 2.9. Statistical Analysis

Statistical analysis was carried out using one-way analysis of variance (ANOVA) and the results are presented as mean ± standard deviation (*n* = 3).

## 3. Results

### 3.1. Determination of Stemness of ASCs

**Figure 1:**
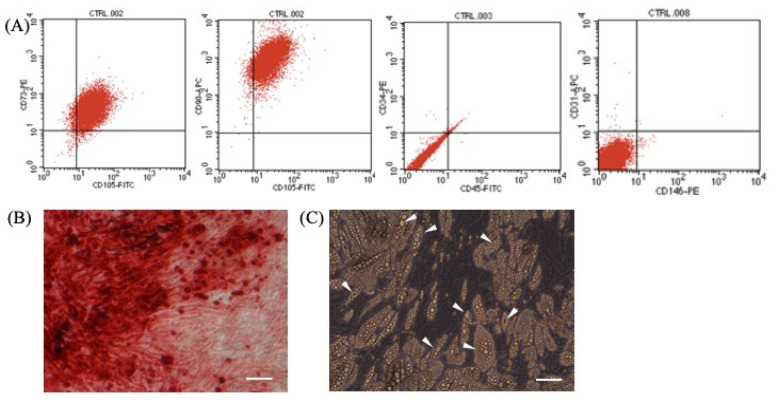
(**A**) Expression of surface antigen markers in isolated adipose-derived stem cells by flow cytometry. (**B**) Osteogenically differentiated ASCs’ deposits of calcium at extracellular matrix confirmed by Alizarin red. (**C**) Adipogenesis is supported by accumulation of lipid droplets (marked by white arrow). Scale bar = 50 μm.

The stemness of ASCs used in the present investigation was confirmed by means of evaluation of definite surface markers and their ability to differentiate into osteogenic and adipogenic lineages, respectively. The ASCs isolated were positive for CD73, CD 90, and CD105 and were negative for CD31, CD34, and CD45 (Figure 1A). Osteogenically differentiated (21 d) ASCs exhibited extracellular calcium deposition that was demonstrated using Alizarin red staining (Figure 1B). Adipogenesis was confirmed by observing the accumulation of lipid droplets under phase microscopy (Figure 1C) in cells following 21 d of adipogenic induction.

### 3.2. Spheroid Formation Dynamics

**Figure 2:**
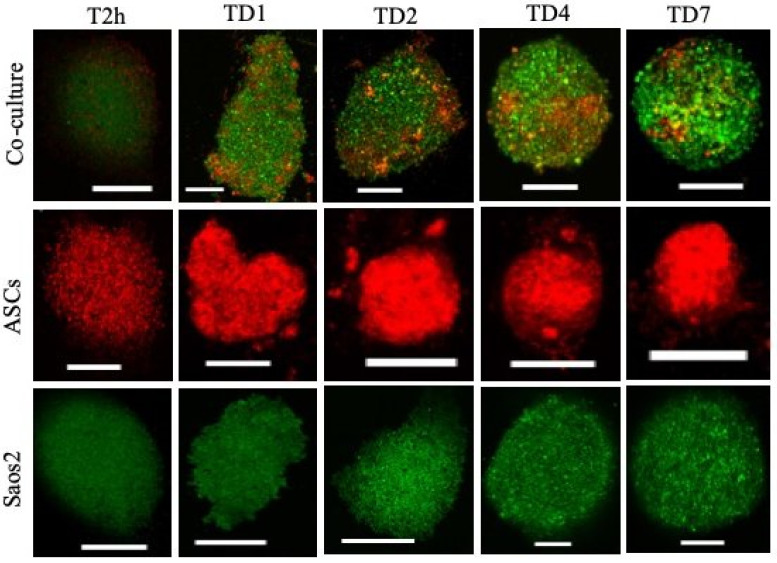
Time-lapse analyses of homo and heterotypic tumorspheres. PKH67 green fluorescent-labeled human Saos 2, PKH26 red fluorescent-labeled ASCs. Scale bar = 250 μm.

The cells formed spheres at the bottom of the plate through gravity-enforced self-assembly within 24 h (Figure 2). The homotypic culture of Saos 2 and heterotypic culture of Saos 2–ASCs became more compact over time as compared to ASCs alone. The compactness of the cell spheres thus generated is dependent on the cell type and the degree of anchorage dependence. The tumorspheres thus generated tended to remain in the same place till the end of the culture (7 days) and were less prone to damage through abrupt movements.

### 3.3. E-Cadherin Expression and Cell–Cell Interactions

**Figure 3:**
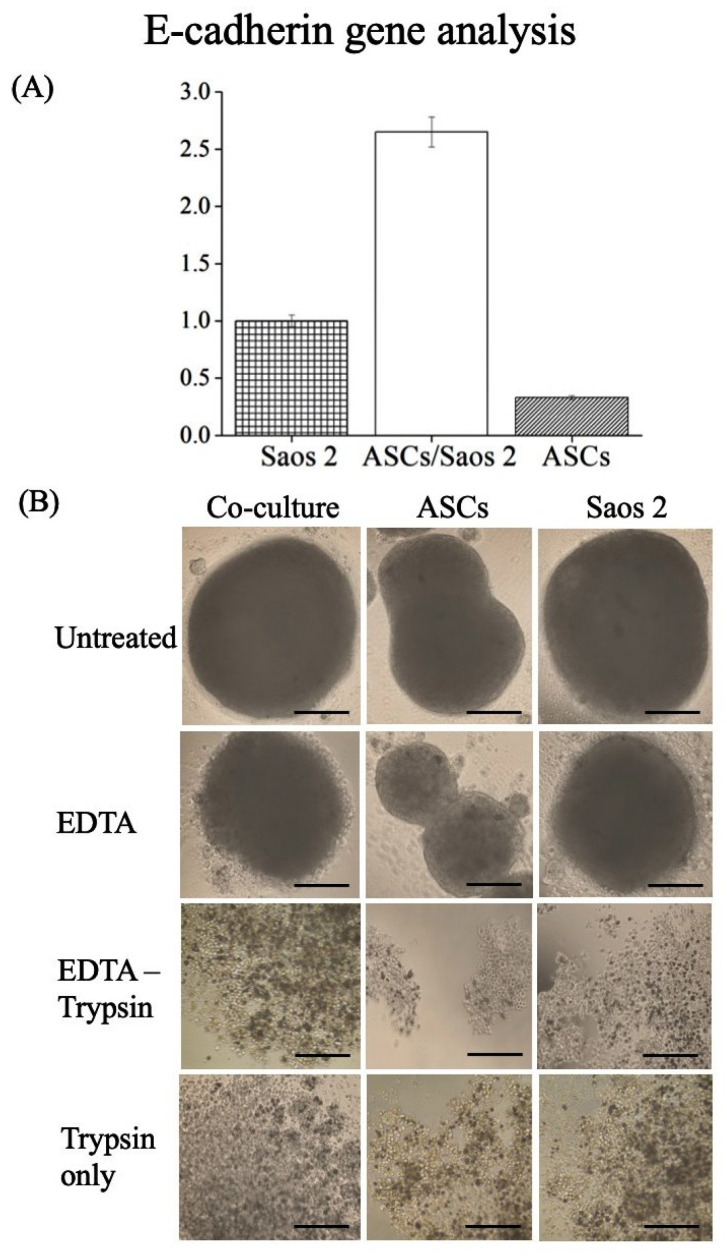
(**A**) Differential expression of E-cadherin by multi-cell spheres. (**B**) Multi-cell sphere separation test in homo- and heterotypic cell spheres [12]. Scale bar = 50 μm.

Gene analysis indicated high levels of E-cadherin expression in the co-culture compared to monoculture (Figure 3A). EDTA is a calcium chelator, and, by chelating the ca-ions, it blocks the activity of calcium-dependent E-cadherin. After EDTA treatment (30 min) of tumorspheres (72 h old), the loss of intercellular connections among cells within the cell spheres and loosened structures could be observed (Figure 3B). Further treatment of tumorspheres with trypsin resulted in the generation of smaller cell masses that were visibly separated from each other (Figure 3B). Only trypsin-treated samples were less fragmented compared to their trypsin–EDTA-treated counterparts.

### 3.4. Mineralization

**Figure 4:**
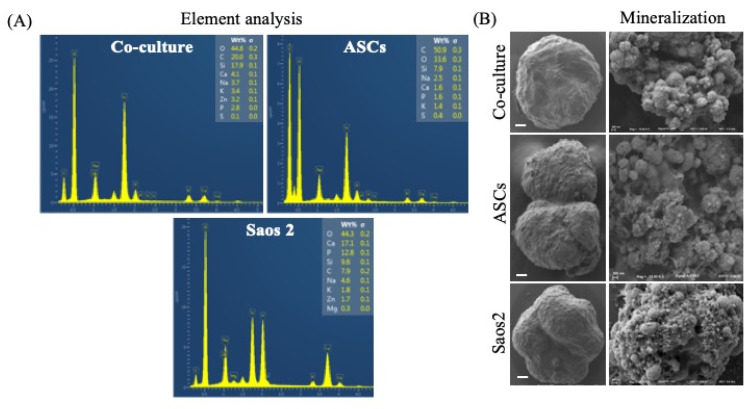
Homo- and heterotypic tumorspheres treated under osteogenic conditions. (**A**) Element analysis of mineralization; (**B**) SEM images of mineralized deposition of calcium phosphate. Scale bar = 50 μm.

Figure 4A shows the EDS spectra and Figure 4B represents the scanning electron micrographs of typical mineralization of tumorspheres. The mineralized hydroxyapatite nodules were observed in all experimental conditions (Figure 4B), which varied from spherical crystals to flake-like morphologies based on cell composition. A mineralized matrix is a prerequisite of native bone; hence, the spheroid culture successfully mimics the physiological niche of the innate tissue.

### 3.5. Drug Resistance and Cytoskeleton Arrangement

**Figure 5:**
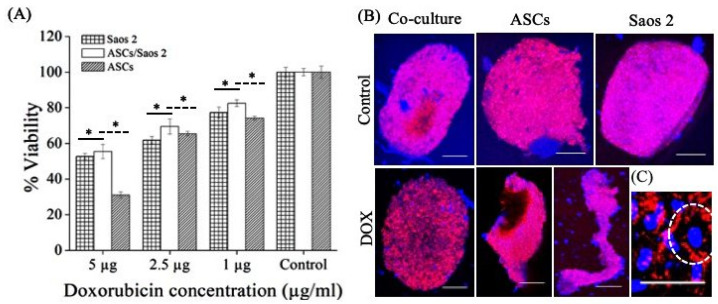
Effect of doxorubicin treatment on (**A**) cell viability (*p* < 0.1) and (**B**) actin arrangement within tumorspheres and ASC spheres. (**C**) Cells exhibiting cortical actin cytoskeleton indicated with white dashed circle. Scale bar = 250 μm.

DOX-induced reduced cell viability was most significant in the ASC homosphere, followed by the Saos 2 tumorsphere (Figure 5A). In contrast, the heterotypic tumorsphere (Saos 2–ASCs) exhibited resistance to DOX-mediated cytotoxicity (*p* < 0.1) (Figure 5A). The homotypic spheres were fragile after treatment and became distorted during pipetting, indicating the loss of cell–cell connection (Figure 5B). The treatment of doxorubicin induced the remodeling of the cytoskeleton, which resulted in the formation of a cortical contractile ring at the periphery of cells (Figure 5C) (observed in homotypic culture of Saos 2). The reference images of the homotypic ASCs and heterotypic tumorsphere are shown in Scheme 1.

### 3.6. Differential Expression of Genes in Homo- and Heterotypic Tumorspheres

**Figure 6:**
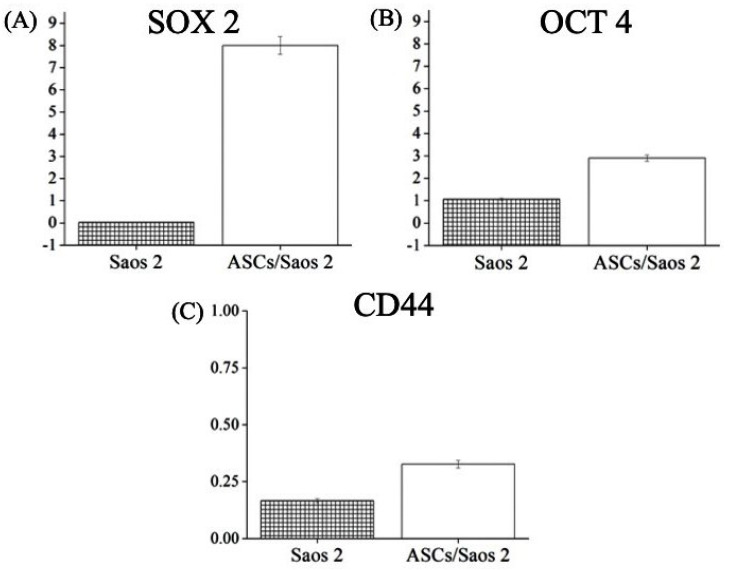
Expression of genes associated with chemoresistance, including (**A**) SOX 2, (**B**) OCT 4, and (**C**) CD44, in cells of homotypic (Saos 2) and heterotypic tumorspheres after doxorubicin treatment.

The expression of genes that are related to drug resistance, such as SOX2, OCT4, and CD44, was quantified in both homo- and heterotypic tumorspheres after doxorubicin treatment. SOX2 was upregulated in both Saos 2 and heterotypic tumorspheres, but more so in the latter (Figure 6A). High expression of OCT 4 and CD44 was also observed in heterotypic tumorspheres compared to homotypic tumorspheres after treatment with doxorubicin (Figure 6A,C).

## 4. Discussion

The crosstalk between stromal and epithelial cells regulates tissue regeneration-development-homeostasis. Recently, the active involvement of mesenchymal stem cells in cancer stroma, which suggests that a tumor is a wound that never heals, has been reported [15]. Tumor cells recruit stem cells including ASCs to heal these wounds [1]. The exosomes secreted by ASCs not only manipulate the behavior of osteosarcoma cells [4] but also have immunomodulatory effects on COVID-19 patients [16]. Hence, the investigation of adipoSIGHT in the therapeutic response is timely and is a prerequisite for risk-free clinical applications of ASCs. The present report seeks to establish a correlation between ASCs and the chemotherapeutic response by employing a realistic 3D in vitro hetero-cellular cancer model.

In time-lapse analyses of the cell spheres, all three phases of spheroid formation, namely initial aggregation, compaction, and spheroid growth, are observed (Figure 2) [17]. The cell–cell adherence is further confirmed by the expression of E-cadherin. E-cadherin is an intercellular adhesion molecule that mediates cell–cell adherence [18]. High expression of E-cadherin is also associated with compact tumorsphere formation, maintenance, and drug response [11]. Increased E-cadherin expression in tumorspheres represents tighter cell–cell contacts, the formation of compact tumorspheres, and long-lasting maintenance. To further confirm the specific role of E-cadherin in cellular crosstalk and the compactness of tumorspheres, its function was blocked using EDTA (a Ca chelator). Upon blocking, the homotypic cell spheres and tumorspheres were disaggregated and the cells escaped from the mass. The cells escaped more readily from homotypic spheroids (i.e., ASCs only) as compared to heterotypic tumorspheres. Based on this, it could be inferred that E-cadherin mediated tight cell–cell connections in spheroids; in particular, heterotypic tumorspheres limit the penetration or diffusion of nutrients and soluble factors, which results in poor infiltration. A similar phenomenon has been reported in osteocarcinoma cells cultured in 3D as compared to their 2D counterparts, indicating the regulatory role of the spatial arrangement of cells in cellular crosstalk [18]. Further, E-cadherin-mediated resistance has been reported in the literature, which confirms its significant role in manipulating the expression of genes involved in drug resistance [19]. Overexpression of E-cadherin is also found in ovarian surface epithelium cells that are located in deep clefts and cysts susceptible to cancerization [20]. Hence, 3D cell culture models, including the one developed in the present study, are a more realistic choice to investigate the clinical outcomes of ASC-mediated therapy (regenerative medicine or therapeutic molecule delivery) in cancer patients.

The scaffold-free nature of tumorspheres makes them highly suitable for investigating the direct effect of exogenous factors added to medium, such as therapeutic molecules. Since doxorubicin (DOX) is employed as a first-line therapy, its effect on homo- and heterotypic tumorspheres can be monitored as a function of concentration. The actin skeleton plays a critical role in cell contact formation in cell spheres [17] and can be used as an indicator of DOX cytotoxicity. The remodeling of the cytoskeleton post-treatment results in the interruption of central stress fibers, causing impaired cell adhesion, which further leads to apoptosis and necrosis [21]. The compact actin and the absence of the cortical contractile ring in heterotypic tumorspheres (Scheme 1) corroborates the finding that mesenchymal stem cells play a significant role in conferring chemoresistance to cancer cells [22].

SOX2 and OCT4 are highly expressed in cancer cells obtained from post-chemotherapy cancer patients compared to their pre-chemotherapy counterparts. SOX2 balances the self-renewal of tumor-initiating cells in osteosarcoma [23]. The knockdown of SOX2 in cancer cells results in the loss of tumorigenicity and stemness in cancer cells [24]. Overexpression of SOX2 inhibits the chemotherapy (cisplatin)-induced cell apoptosis in lung cancer cells [25]. Similar behavior was observed in the present investigation (Figure 6). The high expression in co-cultured cells and the corresponding resistance to doxorubicin treatment are corroborated by previous findings of SOX2-mediated chemotherapeutic resistance in osteosarcoma [18]. CD44 is a transmembrane surface protein; its altered expression is linked to different malignancies [26]. Overexpression of CD44 has been reported in metastatic osteosarcoma and serves as a prognostic marker. CD44 is chiefly a receptor for hyaluronic acid but it also interacts with other niche components such as collagen, osteopontin, growth factors, and cytokines [27]—which together drive both metastasis and chemoresistance. The knockdown of CD44 enhances the sensitivity of osteosarcoma cells to DOX [26]. This supports the present observations that reveal the overexpression of CD44 in heterotypic tumorspheres and their increased spheroid formation capability, stability, and resistance to DOX in vitro. However, the molecular mechanism of CD44-mediated chemoresistance, such as integrin-induced survival signal or overexpression of the ABC transporter, is the subject of ongoing research.

Moreover, CD44-mediated cell–cell and cell–matrix interactions play a vital role in pathophysiological conditions [28]. Thus, the change in the expression of CD44 in tumorspheres directs further investigation towards the screening of mineralized components. The chief inorganic components of bone are minerals (hydroxyapatite, calcium carbonate, calcium fluoride) [11]. The EDS analysis of homotypic spheres and tumorspheres reveals a significant increase in the Zn fraction, which is in line with the mineralization characteristics of patients with osteosarcoma [29]. However, no correlation between Zn content and chemoresistance could be established in the present study; thus, further investigation to determine its potential role in disease progression and drug response is needed.

## 5. Conclusions

In the presence of adipose-derived stem cells, osteosarcoma cells elicit resistance to chemotherapeutics through E-cadherin upregulation. The tighter cell–cell bonding resists the penetration of drugs, while the overexpression of genes related to tumorigenicity, aggressiveness, and chemoresistance increases the tolerance of cells in heterotypic tumorspheres against doxorubicin. These results together improve the understanding of the risks associated with the therapeutic use of adipose-derived stem cells in cancer. They also suggests that serious attention should be paid to the development of stem-cell-targeted therapeutic strategies to overcome chemoresistance.

## Figures and Tables

**Table 1:** Human primer pairs.

Genes	Primer Sequences	Amplicon (bp)	Annealing T (°C)	ID
OCT4	Fw 5′–TACTCCTCGGTCCCTTTCC–3′	167	60	NM_002701.6
	Rw 5′–CAAAACCCTGGCACAAACT–3′			
Sox-2	Fw 5′–ACACCAATCCCATCCACACT–3′	224	60	NM_003106.4
	Rw 5′–GCAAACTCCCTGCAAAGCTC–3′			
CD44	Fw 5′–CGCAGCCTGGGGACTCTG–3′,	117	60	NM_001202555.2
	Rev 5′–CGAGAGATGCTGTAGCGACCA–3′			
E-Cadherin	Fw 5′–AAGAAGCTGGCTGACATGTAC–3′	105	58.8	NM_001317185.2
	Rv 5′–ACCAGCAACGTGATTTCTGCAT–3′			
GAPDH	Fw 5′–AGCCTCAAGATCATCAGCAA–3′	101	-	NM_001289745.2
	Rv 5′–GTCATGAGTCCTTCCACGAT–3′			

## Data Availability

Not applicable.
